# Impact of oral diseases on 12- and 15-year-old children’s quality of life: condition-specific oral health related quality of life analysis

**DOI:** 10.1186/s12903-023-03435-8

**Published:** 2023-10-06

**Authors:** Issarapong Kaewkamnerdpong, Nipaporn Urwannachotima, Piyada Prasertsom, Nuttirudee Charoenruk, Sudaduang Krisdapong

**Affiliations:** 1 Department of Community Dentistry, Faculty of Dentistry, Chulalongkorn University, Bangkok, 10330 Thailand; 2 Department of Health, Bureau of Dental Health, Ministry of Public Health, Nontaburi, 11000 Thailand; 3 Chulalongkorn Business School, Chulalongkorn University, Bangkok, 10330 Thailand; 4 Independent researcher, Bangkok, 10330 Thailand

**Keywords:** Dental caries, Gingival disease, Oral health-related quality of life, Adolescent

## Abstract

**Background:**

Oral diseases can affect children’s quality of life. The aim of the present study was to assess the Condition-Specific (CS) impacts from oral diseases in 12- and 15-year-old Thai population using data from the two consecutive national oral health-related quality of life surveys.

**Methods:**

The oral health–related quality of life surveys were conducted for this study as a part of 6th and 7th national oral health survey. The study sample of 1,066 12- and 815 15-year-olds from 6th national oral health survey; and 556 12- and 351 15-year-olds from 7th national oral health survey were from Bangkok and four regions of Thailand. Oral impacts were assessed by the Child-Oral Impacts on Daily Performances index for 12- and Oral Impacts on Daily Performances index for 15-year-olds. The overall impacts and CS impacts attributed to oral diseases were calculated. The detailed characteristics of the CS impacts were analyzed. Cochran’s Q test and McNemar’s test were used to determine the difference between the proportions of CS impacts of caries, gingival diseases, oral lesions, and malocclusion.

**Results:**

CS impacts-caries were prevalent for both age, followed by gingival disease and oral lesions. CS impacts-caries were the highest in the intensity and extent for both age groups. CS impacts-caries were significantly higher than those of gingival diseases for eating, speaking, relaxing, emotional state, and studying. CS impacts-gingival disease was significantly higher than caries for cleaning in one survey.

**Conclusions:**

CS impacts-caries were the most prevalent and severe among adolescent. Gingival disease was infrequently related to severe impacts on daily performances.

## Background

Oral health is a comprehensive function of individuals’ ability to eat, speak, and perform facial expressions with confidence; but without disease of the craniofacial complex and discomfort [1]. The oral health-related quality of life (OHRQoL) measures were developed to assess consequences of oral diseases on daily life in terms of physical, psychosocial, and social wellbeing [2]. However, most OHRQoL indices are used to assess overall oral impacts, not specific to certain oral diseases. The Oral Impacts on Daily Performances (OIDP) [3] and Child-Oral Impacts on Daily Performances (Child-OIDP) [4] were the instruments used for measuring oral health-related quality of life and they were specifically designed for quantifying condition-specific (CS) impacts attributed to dental caries (CS impacts-caries), gingival disease (CS impacts-gingival disease), oral lesions (CS impacts-lesions), and malocclusion (CS impacts-malocclusion) [5–10].

Condition-specific OHRQoL measures were found to have better discriminative ability compared with generic measures [11] and could be used for specific services planning. High numbers of decayed teeth or severe decay were significantly associated with CS impacts-caries [5–8]. Individuals with extensive calculus and/or gingivitis were more likely to report moderate to high level of CS impacts-gingival disease [9]. Oral lesions, especially recurrent aphthous stomatitis (RAS), can affect children’s quality of life related to daily performance, such as eating, cleaning teeth, and emotional stability [10]. Malocclusion can disturb social interactions and facial expressions among adolescents [12–14]. Thailand, Norway, Germany and the United States had the OHRQoL measures included in the national oral health surveys for school-age children [15–18] and there have been some reports on CS impacts using national data [5–7, 10].

Dental caries and gingival disease are the most common oral diseases among children and adolescents. Worldwide data showed the prevalence around 40% of caries [19–21] and 60–80% of gingival disease in these population (60–80%) [22]. The 6th Thailand National Oral Health Survey (TNOHS) in 2008 reported the prevalence of gingival diseases among 12- and 15-year-old children as high as 82.0 and 86.0% respectively, while the prevalence of dental caries were 56.9 and 66.3%, respectively. The 7th TNOHS in 2013 found the same pattern in both age groups. The prevalence of gingival disease was 70.1 and 78.4%, while that of dental caries was 52.3 and 62.4% for 12- and 15-year-olds, respectively [23, 24]. Therefore, CS OHRQoL analysis from the two consecutive TNOHSs would provide better understanding of how different kinds of oral diseases, particularly dental caries and gingival disease, impacted children’s quality of life. The objectives of this study were to assess the CS impacts attributed to oral diseases, in 12- and 15-year-old Thai population using data obtained from the two consecutives national OHRQoL surveys.

## Methods

### Setting and sampling procedures

The 6th and 7th Thailand National Oral Health Survey (TNOHS) used a stratified multi-stage sampling method which divided Thailand into five strata: Bangkok and four regions (Central, North, Northeast, and South). Four provinces in each region and 4 districts in Bangkok were randomly selected. For each province, one municipal and two rural areas were randomly selected as the ratio of Thai population in municipal and rural areas which is equal to 1:2 respectively [23, 24]. The prevalence of dental caries from the previous Thai national study was used to calculate sample size for the national oral health surveys using margin of error 0.07, 95% confidence interval, design effect of 1.5, and 10% added for unexpected loss. The participants were randomly selected from the five strata across the age ranges from 3 to 89 years old with proportionate to the Thai population size. The 6th and 7th national survey’s sample size was 2,200 12- and 1,742 15-year-olds and 2,312 12- and 1,552 15-year-olds, respectively [23, 24].

Two cross-sectional consecutive oral health–related quality of life surveys as part of the 6th and 7th Thailand National Oral Health Survey were conducted for this study. The survey protocols were approved by the Ethical Committee of the Ministry of Public Health of Thailand. This study focused on the children aged 12 years and 15 years old. Due to limited resources, this study randomly selected a half (two provinces from each region and two sub-districts in Bangkok) from the 6th TNOHS and a quarter (one province from each region and one sub-district in Bangkok) of the sample from the 7th TNOHS. All participants in the selected areas were recruited as the study’s sample in this study and they gave informed consent before the survey. Thus, this study intended to cover 1,100 12-year-old children and 871 15-year-old adolescents from the 6th TNOHS, and 578 12-year-old children and 388 15-year-old adolescents from the 7th TNOHS.

### Data collection

Thai versions of the Child-OIDP [4] and OIDP [3], the instruments that measure oral health-related quality of life, were used to assess oral impacts during the past 3 months for 12-year-olds and past 6 months for 15-year-olds, respectively. The oral impacts were assessed through the difficulties on 8 daily performances: (a) Eating, (b) Speaking, (c) Cleaning teeth, (d) Emotional state, (e) Relaxing/sleeping, (f) Smiling without feeling embarrassment, (g) Studying, and (h) Social contact. [25] The frequency and severity scores were recorded for each performance, ranging from 0 to 3 for the Child-OIDP and 0–5 for the OIDP. For any oral impact, the participants were requested to explain their perceived causes of the impacts [25].

The participants were asked to rate their perceived overall oral health problems on a 4-point Likert scale for the 12-year-olds (none, little, moderate, and much); and 6-point Likert scale for the 15-year-olds (none, very little, little, moderate, much, and very much). The participants were also asked about their perceived dental treatment needs. The interview data were collected after oral examination, by 2 interviewers (dentists or dental hygienists) in each province. Before data collection, the interviewers were well trained and calibrated [25]. Details on the calibration exercises can be found elsewhere [15]. Both TNOHS were conducted by the same survey team and same trainers [23, 24]. 10% of the participants were randomly re-interviewed for the intra-examiner reliability test.

### Data analyses

SPSS Version 22.0 (SPSS, Inc., Chicago, IL, USA) was used for data analysis. The data entry was double checked to ensure accuracy. For the Child-OIDP and OIDP indices, the Intraclass Correlation Coefficient (ICC) was used to determine the intra-examiner reliability. In addition, the Kruskal-Wallis and Mann-Whitney U tests were used to examine the overall impact score across the level of perceived overall oral problems and to compare between two groups of children (the group of children that perceived dental treatment need VS the group of children that did not perceive dental treatment need) [25]. The overall impact scores are expected to be increase as the level of perceived overall oral problems increases. In addition, the overall impact scores should be higher in the group of children that perceived dental treatment need compared to the group of children that did not perceive dental treatment need.

Child-OIDP and OIDP scores were calculated by multiplying the frequency score with the severity score in each performance, ranging from 0 to 9 for the Child-OIDP and 0–25 for the OIDP. The sum of 8 performance scores were divided by the maximum possible score (72 for the Child-OIDP and 200 for the OIDP) and multiplied by 100 (result in an overall score ranging from 0 to 100). The OIDP system includes a question on oral conditions perceived as important causes of the impacts [25]. The answers on the perceived clinical causes were used to calculate the CS impacts relating to that disease. Eight types of CS impacts were calculated: (a) dental caries (perceived causes were toothache, sensitive tooth, hole in tooth, or broken filling), (b) gingival disease (inflamed gums, pain in gums, calculus, or bad breath), (c) oral lesions (RAS, other oral lesions such as herpes, or dry or cracked lips), (d) malocclusion (tooth position), (e) discoloration (tooth color), (f) traumatic injuries (fractured tooth), (g) tooth loss (space due to an extracted permanent tooth), and (h) natural processes (exfoliating primary tooth or erupting permanent tooth [25].

Because a shortcoming in using impact scores was previously revealed [26], the degree of the impacts are presented as intensity and extent. Intensity reflects the severity of the impact, determined by the most severe impact score (9 for the Child-OIDP and 25 for the OIDP) of the 8 performances. Intensity was categorized into six ordinal levels, ranging from none to very severe. Extent was defined as the number of performances with impacts (PWI) showing the scope of oral impacts on daily life, ranging from 0 to 8 PWI [25].

The difference between the proportions of CS impacts-caries, CS impacts-gingival disease, CS impacts-lesions, and CS impacts-malocclusion were determined using Cochran’s Q test, and the difference between the proportions of CS impacts-caries and CS impacts-gingival disease were evaluated using McNemar’s test.

## Results

Total participants comprised 1,066 (96.9% response rate) and 556 (96.2% response rate) 12-year-olds and 815 (93.6% response rate) and 351 (90.5% response rate) 15-year-olds from the 6th and 7th TNOHS, respectively. The intra-examiner reliability analyses indicated very good agreement for the 6th and 7th TNOHS (ICC = 0.75–0.91 for 12- and 0.80–0.92 for 15-year-olds).

Comparing the overall impact scores across the level of perceived overall oral problems by using the Kruskal-Wallis test showed that the median of the scores increases as the level of perceived overall oral problems increases (p < .001). In addition, the overall impact scores were compared across the group of children that perceived dental treatment need versus the group of children that did not perceive dental treatment need by using the Mann-Whitney U tests. Those children that did not perceive dental treatment need got the lower median of the score than children that perceived dental treatment need (p < .001). This indicates that the overall impact scores are consistent to the level of perceived overall oral problems and the current perceived dental treatment need.

### Overall and condition-specific oral impacts

Overall impacts were prevalent for both age groups. More than 80% of the 12- and 15-year-old children from the 6th TNOHS and more than 70% of the 12- and 15-year-old children from the 7th TNOHS had experienced oral impacts on their daily life (Table 1). Among the eight performances assessed, eating was the most affected performance for both age groups from the 6th and 7th TNOHS (64.4 and 53.4% in 12- and 64.0 and 49.0% in 15-year-old children, respectively). Cleaning and emotional state performances ranked second and third with similar percentages. The percentage of CS impacts relating to 8 specific oral diseases are shown in Table 1. The findings from the 4 data sets were quite consistent, that is, CS impacts-caries were the highest (47.8% and 35.3% of 12- and 40.7 and 33.0% for 15-year-olds in the 6th and 7th TNOHS, respectively). The results indicated that the prevalence of CS impacts-caries decreased from the 6th to 7th TNOHS for both age groups. CS impacts-gingival disease and CS impacts-lesions ranked second and third with similar percentages, followed by CS impacts-malocclusion.

Significant differences between the proportions among the four kinds of CS impacts in the 6th and 7th TNOHS were found (Table 2). The results of the intensity analyses revealed that most Thai children with impacts had CS impacts at the very little or little level (Table 2), while the CS impacts of the Thai adolescents mostly were of very little to moderate intensity (Table 3). For the extent of CS impacts, most of the impacts occurred on 1–3 PWI for both age groups (Tables 2 and 3). The highest percentage of CS impacts-lesions occurred on 2 and 3 PWI for 12- and 15-year-old, respectively. Among the four kinds of CS impacts, we compared the percentage of subjects experiencing high intensity (moderate to very severe) and high extent (4–8 PWI) and found that CS impacts-caries were highest for both the intensity and extent, for both age groups (Tables 2 and 3). Significant differences were found (p < .001) for the comparisons of the four kinds of CS impacts from both surveys among the high intensity and high extent. Moreover, the proportions of CS impacts-caries, at high intensity and high extent were significantly higher (p < .001) compared with the proportions of CS impacts-gingival disease at high intensity and high extent.

**Table 1:** Percentage of overall impacts on daily performances and condition-specific (CS) impacts attributed to oral diseases of 12-year-old (N = 1,066) and 15-year-old (N = 815) children from the 6th Thailand National Oral Health Survey (TNOHS) and of 12-year-old (N = 556) and 15-year-old (N = 351) children from the 7th TNOHS.

	12-year-olds (%)		15-year-olds (%)	
	6th survey	7th survey	6th survey	7th survey
Overall impacts	85.2	75.2	83.3	70.1
Performance affected				
Eating	64.4	53.4	64.0	49.0
Speaking	12.7	9.5	15.2	11.1
Cleaning teeth	51.7	44.1	55.3	40.2
Relaxing/sleeping	12.6	11.3	8.8	12.0
Emotional state	49.1	34.5	53.1	41.3
Smiling	28.6	19.2	25.9	21.1
Studying	5.4	6.5	6.0	9.4
Social contact	12.2	12.6	11.5	10.5
CS impacts				
Dental caries	47.8	35.3	40.7	33.0
Gingival disease	26.0	20.0	29.7	19.9
Oral lesions	25.8	19.6	36.4	17.9
Malocclusion	11.7	5.2	13.0	6.8
Discoloration	8.2	1.3	7.9	3.4
Traumatic injuries	1.5	1.3	1.0	1.7
Tooth loss	0.4	0.2	0.4	0.9
Natural process^*^	10.2	6.7	2.1	0.9

^*^Exfoliating primary teeth and erupting permanent teeth

**Table 2:** Intensity and extent of condition-specific oral impacts (CS impacts) attributed to dental caries, gingival disease, oral lesions, and malocclusions among 12-year-old Thai children from the 6th and 7th Thailand National Oral Health Survey (N = 1,066 and 556)

	CS impacts from the 6th survey (%)				CS impacts from the 7th survey (%)			
	Dental	Gingival	Oral	Malocclusion	Dental	Gingival	Oral	Malocclusion
	caries	disease	lesions		caries	disease	lesions	
Prevalence CI for proportion difference^**^	47.8 ^a3^	26.0^b3^ (17.6,25.8)	25.8 ^b3^ (17.7,26.1)	11.7 ^b3^ (32.2,39.7)	35.3 ^a3^	20.0^b3^ (10.0,20.4)	19.6 ^b3^ (10.2,21.0)	5.2 ^b3^ (25.6,34.3)
Intensity								
Very little	9.5	7.0	4.1	2.3	11.7	7.2	3.5	2.5
Little	19.7	10.2	12.8	4.4	9.5	6.9	8.8	1.1
Moderate	9.2	5.4	5.8	1.9	6.7	2.5	3.4	1.1
Severe	7.8	3.0	2.7	2.3	5.4	2.0	1.3	0.3
Very severe	1.6	0.4	0.4	0.8	2.0	1.4	2.5	0.2
Moderate to very severe CI for proportion difference^**^	18.6 ^a3^	8.8^b3^ (7.1,12.4)	8.9 ^b3^ (7.0,12.3)	5.0 ^b3^ (10.9,16.3)	14.1^a3^	5.9^b3^ (4.7,11.6)	7.2 ^b3^ (3.4,10.2)	1.6 ^b3^ (9.2,15.5)
Extent								
1 PWI^*^	17.1	13.7	4.6	7.6	18.9	13.1	6.5	3.8
2 PWI	15.4	6.4	8.1	2.7	8.5	3.2	5.6	1.3
3 PWI	7.1	3.5	7.9	1.1	3.6	2.5	3.8	0.2
4 PWI	4.1	1.7	3.3	0.2	2.7	0.5	2.0	0
5 PWI	2.7	0.2	1.6	0.1	0.5	0.4	1.3	0
6 PWI	1.0	0.4	0.3	0	0.2	0.2	0.5	0
7 PWI	0.4	0.2	0.1	0	0.5	0	0	0
8 PWI	0	0	0	0	0.4	0	0	0
4–8 PWI CI for proportion difference^**^	8.2 ^a3^	2.5^b3^ (3.9,7.5)	5.3 ^b2^ (0.7,5.1)	0.3^b3^ (6.2,9.6)	4.3 ^a3^	1.1^b2^ (1.3,5.2)	3.8 (-1.8,2.9)	0 ^b3^ (2.5,6.1)

^*^PWI = Number of performances with impacts; ^a^Cochran’s Q test, ^b^McNemar’s test; ^1^P < 0.05, ^2^P < 0.01, ^3^P < 0.001; ^**^ 95% CI for proportion difference in prevalence, in moderate to severe intensity, and in 4–8 PWI between Dental caries and other groups (Gingival disease, Oral lesion, and Malocclusion) [p_dental caries_ - p_j_]

**Table 3:** Intensity and extent of condition-specific oral impacts (CS impacts) attributed to dental caries, gingival disease, oral lesions, and malocclusions among 15-year-old Thai children from the 6th and 7th Thailand National Oral Health Survey (N = 815 and 351)

	CS impacts from the 6th survey (%)				CS impacts from the 7th survey (%)			
	Dental	Gingival	Oral	Malocclusion	Dental	Gingival	Oral	Malocclusion
	caries	disease	lesions		caries	disease	lesions	
Prevalence CI for proportion difference^**^	40.7 ^a3^	29.7^b3^ (6.4,15.7)	36.4 (-0.7,9.2)	13 ^b3^ (23.5,31.7)	33 ^a3^	19.9^b3^ (6.6,19.5)	17.9 ^b3^ (8.6,21.4)	6.8 ^b3^ (20.4,31.7)
Intensity								
Very little	9.8	10.1	8.3	3.0	9.4	9.6	3.7	2.3
Little	12.6	8.3	12.4	2.8	6.8	5.7	5.1	2.6
Moderate	15.0	9.2	13.9	4.8	12.2	3.5	7.1	1.1
Severe	2.2	1.1	1.3	1.8	2.6	0.8	1.7	0.8
Very severe	1.1	1.0	0.5	0.6	2.0	0.3	0.3	0
Moderate to very severe CI for proportion difference^**^	18.3^a3^	11.3^b3^ (3.7,10.2)	15.7 (-0.8,5.9)	7.2 ^b3^ (8.0,14.1)	16.8^a3^	4.6^b3^ (7.8,16.5)	9.1^b2^ (2.9,12.4)	1.9 ^b3^ (10.6,18.8)
Extent								
1 PWI^*^	15.8	16.6	4.9	8.8	11.9	13.9	5.6	4.8
2 PWI	11.4	8.0	9.5	3.0	9.4	3.7	4.9	1.7
3 PWI	5.6	2.9	13.5	0.7	6.3	1.2	5.4	0.3
4 PWI	3.5	1.3	5.9	0.5	3.1	0.8	1.1	0
5 PWI	2.3	0.4	2.2	0	1.7	0	0.3	0
6 PWI	1.2	0.4	0.4	0	0.3	0	0	0
7 PWI	0.9	0.1	0	0	0	0	0.3	0
8 PWI	0	0	0	0	0.3	0.3	0.3	0
4–8 PWI CI for proportion difference^**^	7.9^a3^	2.2^b3^ (3.6,7.7)	8.5 (-3.2,2.0)	0.5^b3^ (5.4,9.3)	5.4 ^a3^	1.1^b2^ (1.5,7.0)	2 ^b1^ (0.5,6.3)	0 ^b3^ (2.9,7.8)

^*^PWI = Number of performances with impacts; ^a^Cochran’s Q test, ^b^McNemar’s test; ^1^P < 0.05, ^2^P < 0.01, ^3^P < 0.001

^**^ 95% CI for proportion difference in prevalence, in moderate to severe intensity, and in 4–8 PWI between Dental caries and other groups (Gingival disease, Oral lesion, and Malocclusion) [p_dental caries_ - p_j_]

**Table 4:** Percentage of condition-specific impacts (CS impacts) on the eight daily life performances among 12-year-old (N = 1,066) children from the 6th Thailand National Oral Health Survey (TNOHS) and among 12-year-old (N = 556) children from the 7th TNOHS.

	CS impacts from the 6th survey (%)				CS impacts from the 7th survey (%)			
Performance affected	Dental	Gingival	Oral	Malocclusions	Dental	Gingival	Oral	Malocclusions
	caries	disease	lesions		caries	disease	lesions	
Eating CI for proportion difference^**^	41.3^a3^	6.4^b3^ (31.4,38.2)	20.3^b3^ (16.9,25.0)	1.6 ^b3^ (36.5,42.7)	30.0 ^a3^	3.2^b3^ (22.5,30.9)	14.9^b3^ (10,20)	1.1^b3^ (24.9,32.8)
Speaking CI for proportion difference^**^	3.8^a3^	1.3^b3^ (1.2,3.7)	7.3^b3^ (-5.5,-1.6)	0.7 ^b3^ (1.8,4.4)	1.6 ^a2^	1.1 (-0.9,2)	2.7 (-0.7,2.9)	0.4^b1^ (0,0.3)
Cleaning teeth CI for proportion difference^**^	16.7^a3^	16.5 (-3.0,3.3)	20.4 ^b1^ (-7.1,-0.2)	2.1 ^b3^ (12.2,17.1)	10.3 ^a3^	13.1 (-6.7,1)	11.9 (-5.4,2.2)	2.0^b3^ (5.4,11)
Relaxing/sleeping CI for proportion difference^**^	9.4 ^a3^	2.0^b3^ (5.5,9.3)	1.5^b3^ (6.0,9.7)	0.1 ^b3^ (7.5,11.0)	4.3 ^a3^	0.5^b3^ (1.9,5.6)	2.9 (-0.7,3.6)	0^b3^ (2.5,6.1)
Emotional state CI for proportion difference^**^	24.1^a3^	11.2^b3^ (9.7,16.1)	13.4^b3^ (7.3,14.1)	3.5^b3^ (17.7,23.4)	12.4 ^a3^	5.8^b3^ (3.1,10.1)	9.0 (-0.3,7.1)	0.4 ^b3^ (9.2,14.8)
Smiling CI for proportion difference^**^	3.5 ^a3^	4.2 (-2.4,0.9)	2.9 (-0.9,2.1)	8.6 ^b3^ (-7.2,-3.1)	3.6	3.6 (-2.2,2.2)	2.2 (-0.6,3.5)	2.9 (-1.4,2.9)
Studying CI for proportion difference^**^	3.9 ^a3^	1.3^b3^ (1.4,3.9)	1.0^b3^ (1.7,4.1)	0.1^b3^ (2.6,5.0)	2.7 ^a3^	0.4^b2^ (0.8,3.8)	1.3 (-0.3,3.2)	0^b3^ (1.3,4.1)
Social contact CI for proportion difference^**^	5.2 ^a3^	5.3 (-2,1.8)	1.0 ^b3^ (2.7,5.6)	1.0 ^b3^ (2.6,5.6)	2.9 ^a3^	4.5 (-0.6,3.9)	1.6 (-0.5,3)	0.2^b3^ (1.2,4.2)

^a^Cochran’s Q test, ^b^McNemar’s test; ^1^P < 0.05, ^2^P < 0.01, ^3^P < 0.001

**95% CI for proportion difference in each performance affected between Dental caries and other groups (Gingival disease, Oral lesion, and Malocclusion) [p_dental caries_ - p_j_]

**Table 5:** Percentage of condition-specific impacts (CS impacts) on the eight daily life performances among 15-year-old (N = 815) children from the 6th Thailand National Oral Health Survey (TNOHS) and among 15-year-old (N = 351) children from the 7th TNOHS.

	CS impacts from the 6th survey (%)									CS impacts from the 7th survey (%)						
Performance affected	Dental		Gingival		Oral		Malocclusions			Dental		Gingival		Oral	Malocclusions	
	caries		disease		lesions					caries		disease		lesions		
Eating CI for proportion difference^**^		35.7 ^a3^		6.0^b3^ (25.9,33.3)		31.5 (-0.7,9)		2.0 ^b3^ (30.2,37.1)	29.3^a3^		3.1^b3^ (20.9,31.3)		11.7^b3^ (11.6,23.7)			0.9^b3^ (23.4,33.3)
Speaking CI for proportion difference^**^		3.4 ^a3^		1.6^b2^ (0.4,3.3)		11.0^b3^ (-10,-5.1)		0.5^b3^ (1.6,4.3)	1.7^a1^		1.7 (-2.1,2.1)		2.6 (-3.1,1.4)			0 ^b1^ (0.1,3.3)
Cleaning teeth CI for proportion difference^**^		12.3 ^a3^		18.5^b3^ (-9.8,-2.7)		30.6 ^b3^ (-22.2,-14.3)		2.5 ^b3^ (7.3,12.3)	11.4^a3^		13.1 (-6.7,3.3)		11.7 (-5.2,4.7)			1.4^b3^ (6.3,13.6)
Relaxing/sleeping CI for proportion difference^**^		7.2 ^a3^		1.3^b3^ (4,7.7)		1.0^b3^ (4.4,8.1)		0 ^b3^ (5.4,9)	5.1^a3^		0.9^b2^ (1.6,6.9)		1.1^b2^ (1.3,6.7)			0 ^b3^ (2.7,7.5)
Emotional state CI for proportion difference^**^		23.2 ^a3^		14.7^b3^ (4.7,12.2)		22.0 (-3,5.4)		3.4 ^b3^ (16.5,22.9)	17.7^a3^		5.7^b3^ (7.3,16.5)		10.3^b2^ (2,12.8)			1.1^b3^ (12.2,20.7)
Smiling CI for proportion difference^**^		2.8 ^a3^		4.8^b1^ (-3.8,-0.1)		2.6 (-1.9,1.4)		9.4 ^b3^ (-8.9,-4.2)	2.3^a2^		4.3 (-4.6,0.7)		1.1 (-0.9,3.2)			4.8 (-0.3,5.4)
Studying CI for proportion difference^**^		4.8 ^a3^		0.6^b3^ (2.6,5.7)		0.7^b3^ (2.5,5.6)		0.1^b3^ (3.1,6.2)	3.7^a2^		0.6^b2^ (0.9,5.4)		2.0 (-0.6,4)			0.6^b2^ (0.9,5.4)
Social contact CI for proportion difference^**^		4.9 ^a3^		4.0 (-1.1,2.8)		2.0^b3^ (1.2,4.7)		0.3^b3^ (2.3,5.6)	3.4^a2^		1.1 (-0.1,4.6)		1.4 (-0.4,4.4)			0.3 ^b2^ (1,5.2)

^a^Cochran’s Q test, ^b^McNemar’s test; ^1^P < 0.05, ^2^P < 0.01, ^3^P < 0.001

**95% CI for proportion difference in each performance affected between Dental caries and other groups (Gingival disease, Oral lesion, and Malocclusion) [p_dental caries_ - p_j_]

Detailed characteristics of CS impacts in relation to the eight daily performances are shown in Table 4. Out of the eight performances, CS impacts on 4 to 5 performances were mostly related to caries for both age groups in the 6th and 7th TNOHS. For 12-year-olds, caries was the main cause of an impact on eating, relaxing, emotional state, and studying, while social contact was mostly impacted by gingival disease, and speaking by oral lesions for both surveys. For the 15-year-olds, a similar pattern was observed. The performances mostly impacted by dental caries were eating, relaxing, emotional state, studying, and social contact, while impacts on speaking and smiling were mostly related to oral lesions and malocclusion respectively. Comparing CS impacts-caries and CS impacts-gingival disease, CS impacts-caries were significantly higher than that of gingival diseases for the five performances, eating, speaking, relaxing, emotional state, and studying for the 12-year-olds in both surveys. For the other three performances, namely, on cleaning, smiling, and social contact, no significant differences were found between the proportions of CS impacts-caries and CS impacts-gingival disease. CS impacts-gingival disease were not significantly higher compared with the proportions of CS impacts-caries on any performance. A similar pattern was observed for the 15-year-olds. CS impacts-caries were significantly higher than that of gingival diseases for the five performances. No significant differences were found between the proportions of CS impacts-caries and CS impacts-gingival disease in smiling and social contact performances. CS impacts-gingival disease were found to be significantly higher than that of caries for cleaning performance (p < .01) in only the 6th TNOHS (Table 5).

**Fig. 1:**
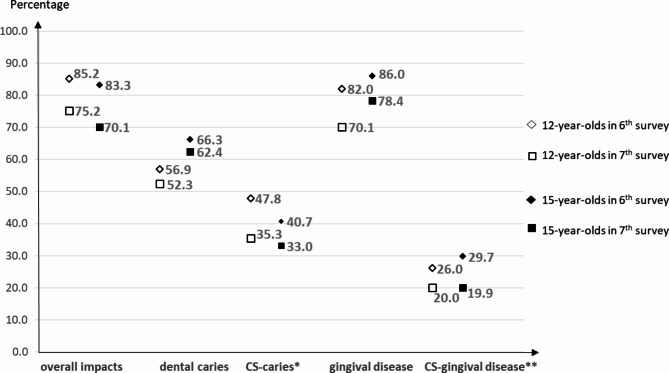
Changes in the prevalence of dental caries and gingival disease and their oral impacts among 12- and 15-year-old Thai children from the 6st and 7nd Thailand National Oral Health Surveys *Condition-Specific impacts attributed to dental caries **Condition-Specific impacts attributed to gingival disease

The comparison between both surveys (Fig. 1) showed that the overall impacts decreased for both age groups. The prevalence of CS impacts-caries and CS impacted-gingival diseases has decreased from the 6th to 7th national oral health survey. However, the reductions in dental caries were much less than those of gingival disease. In the 12-year-old group, the prevalence of dental caries decreased from 56.9 to 52.3%, while that of gingival disease decreased from 82.0 to 70.1%. The same pattern was also observed in the 15-year-old group, where although gingival disease reduced more than dental caries, CS impacts-gingival disease reduced less than those of dental caries.

This is the first study that compared the detailed characteristics of CS impacts attributed to specific oral diseases from two consecutive national surveys. Although the prevalence of gingival disease was higher compared with caries worldwide including in Thailand [19–22], the present study showed that caries affected OHRQoL more than gingival disease did. Thus, if oral health service planning was based on clinical measures; gingival disease would be considered the most important disease among children and adolescents. However, this study has demonstrated that the prevalence and proportions of severe impacts were related more to dental caries compared with gingival disease. Therefore, the current study’s findings suggest that oral health policy aiming at the reduction of dental caries, rather than gingival disease, should result in improved OHRQoL in children and adolescents.

Our findings that the reductions in caries and gingival disease prevalence were not accompanied by similar reductions in their respective CS impacts supports our other findings indicating that dental caries account for the oral impacts on quality of life more than gingival diseases do. These findings also suggest oral health policy that routine surveys would be required to monitor the changes in the population’s OHRQoL pattern in relation to changes in oral diseases. The prevalence of CS impacts-caries comprised approximately half of the prevalence of overall impacts for both age groups in the 6th and 7th TNOHS. In contrast, the CS impacts-gingival disease and oral lesions were markedly lower. However, the results of this study were not consistent with those of a previous study conducted at a provincial level that reported a similar prevalence between CS impacts-caries and CS impacts-gingival disease [8]. Children in rural areas might have poorer oral hygiene leading to higher CS impacts-gingival disease, compared with the national representative sample. In that provincial study, Kaewkamnerdpong and Krisdapong [8] also showed that children in public rural schools were more likely to report CS impacts-gingival disease compared with children in public urban schools. Krisdapong et al. [9] analysed TNOHS data and found that children living in other regions had higher odds for CS impacts-gingival disease compared with children living in Bangkok.

The importance of CS impacts-caries was confirmed through the analyses on intensity and extent. The highest proportions of CS impacts-caries were moderate to very severe intensity and 4–8 PWI for 12- and 15-year-old children. Moreover, among the 8 performances, the prevalence of CS impacts-caries was the highest for 5 performances in both age groups. Further analyses on the four main types of CS impacts in relation to the eight daily performances confirmed that dental caries was the main potential cause of impacts on these performances. These findings are comparable to a previous study showing that a sensitive tooth and toothache impacted most of the 8 daily performances [27]. These findings suggest that to improve quality of life of Thai school-age children, oral health services should prioritize treating dental caries.

The CS impacts-gingival disease were less important compared with those of dental caries. In addition to their relative low prevalence, the CS impacts-gingival disease were the least severe and least extensive impacts, compared with those of dental caries and oral lesions. This is likely because the CS impacts-gingival disease involve little to no pain, while the CS impacts-caries often results from caries associated pain. These findings are similar to those of previous studies indicating the relatively uncommon and less severe CS impacts-gingival disease in school-age children [6, 24, 26]. Nevertheless, the present study found that the importance of gingival disease was related to a specific performance, i.e. cleaning teeth, where the percentages of CS impacts-gingival disease were higher than that of dental caries.

In addition, our study revealed that oral lesion is another disease, in addition to dental caries, that severely impact quality of life. RAS considerably impacts children’s quality of life [4, 17]. The present study’s findings that CS impacts-lesions related specifically to speaking and cleaning for both age groups, agreed with those of a previous study [10]. However, oral ulcers are usually neglected when evaluating CS impacts on OHRQoL. For example, oral ulcers examination is not included in the survey, which may be because oral ulcers are an acute short-term condition. Our findings suggest that if the goals of oral health services are the improvement of quality of life, more attention should be given to oral lesions, particularly in the children and adolescent age group. When analyzing malocclusion-related impacts, we found that CS impacts-malocclusion in the 15-year-old group contributed to approximate 10% of the overall impacts, thus, orthodontic treatment might be needed to minimize these impacts. However, the results of this study were not consistent with those of previous studies in the United Kingdom that reported the prevalence of CS impacts-malocclusion in adolescents were approximately 21% [12, 13]. Esthetic concerns might be culturally relevant. However, malocclusion examination is not included in Thailand’s survey. The difference in CS impacts between countries might be explained if oral status data of the two countries were available.

Our study demonstrated the benefit of using specific OHRQoL measures. While the analysis of overall impacts provides a broad picture of oral disease consequences impacting quality of life [15–18], CS impacts analyses show which oral diseases contributed to a decrease in quality of life and to what degree. The results on overall impacts have limitations on oral health services planning, whereas CS impacts provide information for planners on which kinds of treatment are needed for the population. However, the limitation of the study, the cross-sectional design could not establish the temporal relationships between parameters. Longitudinal studies are needed to clarify these issues.

## Conclusions

CS impacts-caries were the most common, contributing to half of the overall impacts, followed by CS impacts-gingival disease and oral lesions. The impacts on most daily performances were related to dental caries, while cleaning and speaking were generally related to oral lesions. Children and adolescents with impacts of moderate to very severe intensity and higher PWI were mainly related to dental caries. Gingival disease was infrequently related to severe impacts on daily performances.

## Data Availability

The data that support the findings of this study are available from Bureau of Dental Health, Department of Health, Ministry of Public Health, Thailand, but restrictions apply to the availability of these data, which were used under license for the current study, and so are not publicly available. Data analysis during this study are however available from the corresponding author upon reasonable request and with permission of Bureau of Dental Health, Department of Health, Ministry of Public Health, Thailand.

## List of abbreviations

CS: Condition-Specific
OHRQoL: Oral Health-Related Quality of Life
OIDP: Oral Impacts on Daily Performances
Child-: OIDP
TNOHS: Thailand National Oral Health Survey
CS: OHRQoL
